# The first year of the global Enhanced Gonococcal Antimicrobial Surveillance Programme (EGASP) in Bangkok, Thailand, 2015-2016

**DOI:** 10.1371/journal.pone.0206419

**Published:** 2018-11-09

**Authors:** Pachara Sirivongrangson, Natnaree Girdthep, Wichuda Sukwicha, Prisana Buasakul, Jaray Tongtoyai, Emily Weston, John Papp, Teodora Wi, Thitima Cherdtrakulkiat, Eileen F. Dunne, EGASP Thailand Workgroup

**Affiliations:** 1 Department of Disease Control, Thailand Ministry of Public Health, Nonthaburi, Thailand; 2 Thailand Ministry of Public Health–U.S. Centers for Disease Control and Prevention Collaboration, Nonthaburi, Thailand; 3 Bangrak STIs Center, Bureau of AIDS, TB, and STIs, Bangkok, Thailand; 4 Division of STD Prevention, U.S. Centers for Disease Control and Prevention, Atlanta, Georgia, United States of America; 5 Department of Reproductive Health and Research, World Health Organization, Geneva, Switzerland; 6 Division of HIV/AIDS Prevention, U.S. Centers for Disease Control and Prevention, Atlanta, Georgia, United States of America; Emory University School of Medicine, UNITED STATES

## Abstract

Antimicrobial-resistant *Neisseria gonorrhoeae* (NG) infection is a global public health threat, and there is a critical need to monitor patterns of resistance and risk factors. In collaboration with the World Health Organization (WHO), the U.S. Centers for Disease Control and Prevention (CDC), and the Thailand Department of Disease Control (DDC), Ministry of Public Health (MoPH) implemented the first Enhanced Gonococcal Antimicrobial Surveillance Programme (EGASP) in November 2015. Men presenting with urethritis at two clinical settings in Bangkok, Thailand (Bangrak Hospital [BH] and Silom Community Clinic @TropMed [SCC @TropMed]) provided demographic and behavioral information and had a urethral swab for Gram’s stain and NG culture collected. The NG isolates were evaluated for antimicrobial susceptibility by the Epsilometer test (Etest) to determine minimum inhibitory concentrations (MICs) for cefixime (CFM), ceftriaxone (CRO), azithromycin (AZI), gentamicin (GEN), and ciprofloxacin (CIP). From November 2015 –October 2016, 1,102 specimens were collected from 1,026 symptomatic men; 861 (78.1%) specimens were from BH and 241 (21.9%) specimens were from SCC @TropMed. Among the 1,102 specimens, 582 (52.8%) had intracellular Gram-negative diplococci and 591 (53.6%) had NG growth (i.e., NG infection); antimicrobial susceptibility testing (AST) was performed on 590 (99.8%) NG isolates. Among all symptomatic men, 293 (28.6%) had sex with men only, 430 (41.9%) were ages 18–29 years, 349 (34.0%) had antibiotic use in the last 2 weeks, and 564 (55.0%) had NG infection. Among 23 men with repeat NG infection during this first year of surveillance, 20 (87.0%) were infected twice, 2 (8.7%) were infected three times, and 1 (4.3%) was infected more than four times. All NG isolates were susceptible to CFM and CRO, and had MICs below 2 μg/mL for AZI and below 16 μg/mL for GEN. Overall, 545 (92.4%) isolates were resistant to CIP. This surveillance activity assessed individual patients, and included demographic and behavioral data linked to laboratory data. The inclusion of both individual and laboratory information in EGASP could help identify possible persistent infection and NG treatment failures. Expansion of EGASP to additional global settings is critical to assess trends and risk factors for NG, and to monitor for the emergence of resistance.

## Introduction

*Neisseria gonorrhoeae* (NG) is a common, global sexually transmitted infection that can have serious complications. There is increasing concern about the development of antimicrobial-resistant NG to extended-spectrum cephalosporins, which are the main drugs recommended for primary NG infection treatment globally [1–5]. Several countries have documented treatment failure to cephalosporins and/or elevated Minimum Inhibitory Concentrations (MICs) to ceftriaxone (MIC ≥0.25 μg/mL) [6–19] and widespread cephalosporin- resistant NG infection is anticipated to emerge, including emergence in Western Pacific and South-East Asia region countries [20]. The current research and development pipeline for new treatments of gonorrhea is very limited, with only three new antimicrobial products in various stages of clinical development [21].

As a result, surveillance is crucial for detecting and monitoring antimicrobial resistance (AMR). The WHO’s Gonococcal Antimicrobial Surveillance Programme (GASP), a global laboratory network, has monitored gonococcal antimicrobial susceptibility data since 1992; Thailand has been collecting these data since the inception of the program. There have been no isolates with reduced susceptibility to ceftriaxone or cefixime to date through GASP [22] or in other studies [23–27] in Thailand. However, two clinical isolates have recently been found to have elevated MICs to cephalosporins (e.g., cefixime and/or ceftriaxone) [28]. In addition to GASP, several other surveillance systems have been implemented in specific countries or regions to monitor trends for antimicrobial resistant NG. Each of these allow for the analysis of epidemiological and laboratory data including the Centers for Disease Control and Prevention’s (CDC) Gonococcal Isolate Surveillance Project (GISP) in the United States (U.S.) [29], Public Health England’s Gonococcal Resistance to Antimicrobials Surveillance Programme (UK-GRASP) in the United Kingdom [30], and the Australian Gonococcal Surveillance Programme (AGSP) [31].

The WHO GASP program collects susceptibility data from participating countries. Countries collect isolates through existing infrastructure and laboratory methods which vary across participating countries, making it difficult to compare susceptibility patterns across countries. GASP does not include demographic and behavioral data or information on unique individuals with NG infection, limiting its ability to identify populations at increased risk for antimicrobial-resistant NG. The Enhanced Gonococcal Antimicrobial Surveillance Programme (EGASP) was implemented as a collaboration between the WHO and CDC to strengthen surveillance by standardizing data and laboratory collection for antimicrobial-resistant NG [32]. Selected surveillance sites in Bangkok, Thailand became the first setting to begin EGASP in November 2015. This manuscript describes the first year of EGASP surveillance in Thailand during November 2015 –October 2016 and are important for early detection of NG resistant strains and informing treatment options.

## Methods

### Study population and data collection

EGASP in Thailand was implemented in two clinical settings in Bangkok, Thailand: Bangrak Hospital (BH) and Silom Community Clinic @TropMed (SCC @TropMed). BH is a public clinic that provides diagnosis and treatment of sexually transmitted infections (STI) to males through two separate clinical settings: a) a General Male Clinic (GMC) for heterosexual men and bisexual men, and b) a Male Health Clinic (MHC) for transgender women (TGW), and men who have sex with men (MSM). SCC @TropMed is a research setting that provides voluntary HIV and STI counseling and testing to MSM and TGW.

Since November 2015, all men (male at birth) attending these clinical settings were routinely asked about symptoms of urethritis (discharge or dysuria). If urethral symptoms were reported, men were enrolled into EGASP. Select demographic, behavioral, and clinical data were obtained in addition to the collection of two urethral swab specimens. One specimen was streaked on an agar plate with selective media (i.e., an in-house modified Thayer-Martin media), and a separate swab was collected and smeared on a slide for Gram’s stain. Culture plates were kept in a 5% CO_2_ incubator, and transported as needed by candle jar.

This activity was determined to be non-research by CDC’s, Division of STD Prevention Office of the Director and the National Center for HIV/AIDS, Viral Hepatitis, STDs, and TB Office of the Director. The Thailand Ministry of Public Health reviewed and approved the protocol as routine disease surveillance activity. The activity was considered public health practice and routine surveillance. There were no personal identifiers collected in the database.

### Isolate identification and antimicrobial susceptibility testing

Culture confirmation and nucleic acid amplification testing for *N*. *gonorrhoeae* are not widely performed in local hospitals in Thailand. However, the current Thai National Guidelines for the Treatment of STDs recommends both a syndromic and laboratory diagnosis for gonorrhea [4]. Laboratory criteria includes (1) presence of Gram-negative intracellular diplococci by gram stain as a basic laboratory investigation and (2) a culture confirmatory test for *N*. *gonorrhoeae*.

The two reference laboratories chosen to participate in EGASP surveillance had the capacity to perform both gram stain and culture confirmation on *N*. *gonorrhoeae* isolates. Additionally, prior to EGASP implementation, all laboratory personnel underwent a training from CDC’s Division of STD Prevention to review the proper techniques for gram stain, culture confirmation, and antimicrobial susceptibility testing. BH sent specimens to the BH Laboratory and SCC @TropMed sent specimens to the Hospital of Tropical Medicine Microbiology laboratory for confirmatory testing.

All laboratories participated in regular Internal Quality Control (IQC) measures and twice-yearly External Quality Assessment (EQA) activities coordinated with CDC’s Division of STD Prevention. Presumptive identification of *N*. *gonorrhoeae* was based on the following criteria: (i) growth of typical-appearing colonies on a selective medium such as Thayer-Martin at 35°C–36.5°C in 5% CO_2_, (ii) a positive oxidase test, (iii) the observation of Gram-negative diplococci in stained smears, and (iv) a positive superoxol (catalase) test. Confirmatory identification was performed by using the Rapid Micro Carbohydrate Test (RMCT), which is the method to test the bacterium’s ability to produce acid from sugar (2% glucose, 2% maltose, and 2% sucrose). After an isolate was confirmed as NG, a pure culture of each isolate was suspended in skim milk or cryopreservation beads and was stored at -70°C in duplicate for batch antimicrobial susceptibility testing (AST) and long-term storage.

Frozen gonococcal isolates were thawed and sub-cultured on chocolate agar (Oxoid, Basingstoke, UK), then incubated at 35–36°C, 5% CO_2_ for 18–20 hours until pure cultures were obtained. The pure NG colonies were then suspended in a Mueller Hinton broth (Difco, Sparks, Maryland, USA) adjusted to a turbidity equivalent to 0.5 McFarland BaSO_4_ standard. A dry swab was inoculated into the NG suspension, streaked on GC agar (Oxoid, Basingstoke, UK), and evaluated for antimicrobial susceptibility by the Epsilometer test (Etest, bioMérieux Inc, Durham, North Carolina, USA) to determine minimum inhibitory concentrations (MICs) for cefixime (CFM), ceftriaxone (CRO), azithromycin (AZI), gentamicin (GEN), and ciprofloxacin (CIP). Etest was chosen to perform AST for EGASP after assessing it was a suitable alternative to agar dilution, which is the gold standard for AST [33–35]. Etest was performed on three quality control strains including: (i) WHO P, (ii) ATCC 49226, and (iii) WHO K or WHO L (alternated use) at each run. The GC plates with Etest strips were then incubated at 35–36°C in 5% CO_2_ for 20–24 hours until growth. Results from the Quality Control strains were reported monthly with the antimicrobial susceptibility data. As there are no established or standard alert values, we created alert MIC value criteria: CRO (≥0.125 μg/mL), CFM (≥0.25 μg/mL), AZI (≥2 μg/mL), and GEN (≥16 μg/mL). This alert system was implemented for early response and to initiate repeat testing, and it is similar to other alert values used in the U.S. GISP [29]. Although ≥32 μg/mL is the MIC alert value most often used for GEN [36], a value of ≥16 μg/mL was used in Thailand. The MIC breakpoint for CIP was ≥1.0 μg/mL; this breakpoint for NG isolates was classified by Clinical and Laboratory Institute Standards (CLSI) [37].

If any isolate had an MIC value at or above an alert value (i.e., an initial alert), the isolate was retested using the same methodology within 5 working days. If the isolate was re-confirmed with an MIC value at or above the alert value, this information was reported immediately to in-country EGASP staff as well as CDC and WHO as the final alert value.

### Data analysis

We conducted descriptive statistics using counts and proportions to describe characteristics of specimens, NG isolates, and men enrolled in EGASP in addition to gonococcal antimicrobial susceptibility data. Descriptions of men with repeat NG infection include only men who had documented treatment at their initial visit. All analyses were performed using STATA Version 12.0 (StataCorp, College Station, TX, USA).

## Results

From November 2015 –October 2016, 3,143 men attended BH and 1,626 men attended SCC @TropMed. During this period, 1,026 (21.5%) men with urethritis visited one of the participating clinics and 1,102 specimens were collected as a part of EGASP Thailand. Most specimens (78.1%) were from BH. Among the 1,102 specimens, 582 (52.8%) were Gram stain positive for intracellular diplococci and 591 (53.6%) isolates were culture positive for NG infection; 511 (46.4%) specimens were neither Gram stain positive nor culture positive. After identification of NG infection, one isolate was found to be contaminated and was not further characterized (Table 1).

**Table 1:** Characteristics of collected urethral specimens from men with urethritis, EGASP Thailand, November 2015 –October 2016 (n = 1,102).

Characteristics	N (%)			
	BH		SCC	Total
	GMC	MHC		
**Number of urethral specimens**	695 (63.1)	166 (15.1)	241 (21.9)	1,102 (100.0)
**Gram stain positive**	373 (53.7)	112 (67.5)	97 (40.2)	582 (52.8)
**NG culture positive**	372 (53.5)	114 (68.7)	105 (43.8) ^†^	591 (53.6)
**Number with AST Results**	371 (99.7) ^‡^	114 (100.0)	105 (100.0)	590 (99.8)
*Alert MIC—Initial	1 (0.3)	0 (0.0)	3 (2.9)	4 (0.7) ^§^
*Alert MIC—Final	0 (0.0)	0 (0.0)	0 (0.0)	0 (0.0)

**Abbreviations: BH** = Bangrak Hospital, **SCC** = Silom Community Clinic @TropMed, **GMC** = General Male Clinic, **MHC** = Male Health Clinic, **AST** = Antimicrobial Susceptibility Test, **MIC** = Minimum Inhibitory Concentration

*Alert MIC criteria: Ceftriaxone MIC ≥ 0.125 μg/mL, Cefixime MIC ≥ 0.25 μg/mL, Azithromycin MIC ≥ 2 μg/mL, and Gentamicin MIC ≥ 16 μg/mL

^†^1 case had missing culture/identification results

^‡^1 case was found to be contaminated before AST was performed

^§^3 cases with initial alert MIC to GEN and 1 case with initial alert MIC to AZI

The majority of NG infections were treated according to the Thailand National Guidelines for Sexually Transmitted Diseases (STD) Treatment [4] with the recommended Ceftriaxone 250 mg IM (98.5% of cases) or Cefixime 400 mg PO (0.7% of cases); few cases (0.7%) received Azithromycin 2 gm PO only. In addition, the majority of cases (98.5%) were co-treated with another antibiotic for non-gonococcal urethritis: Azithromycin 1 gm PO (42.1% of cases), Azithromycin 2 gm PO (1.9% of cases), Doxycycline 100 mg PO (28.8% of cases), or another non-specified antibiotic (25.5% of cases).

**Fig 1:**
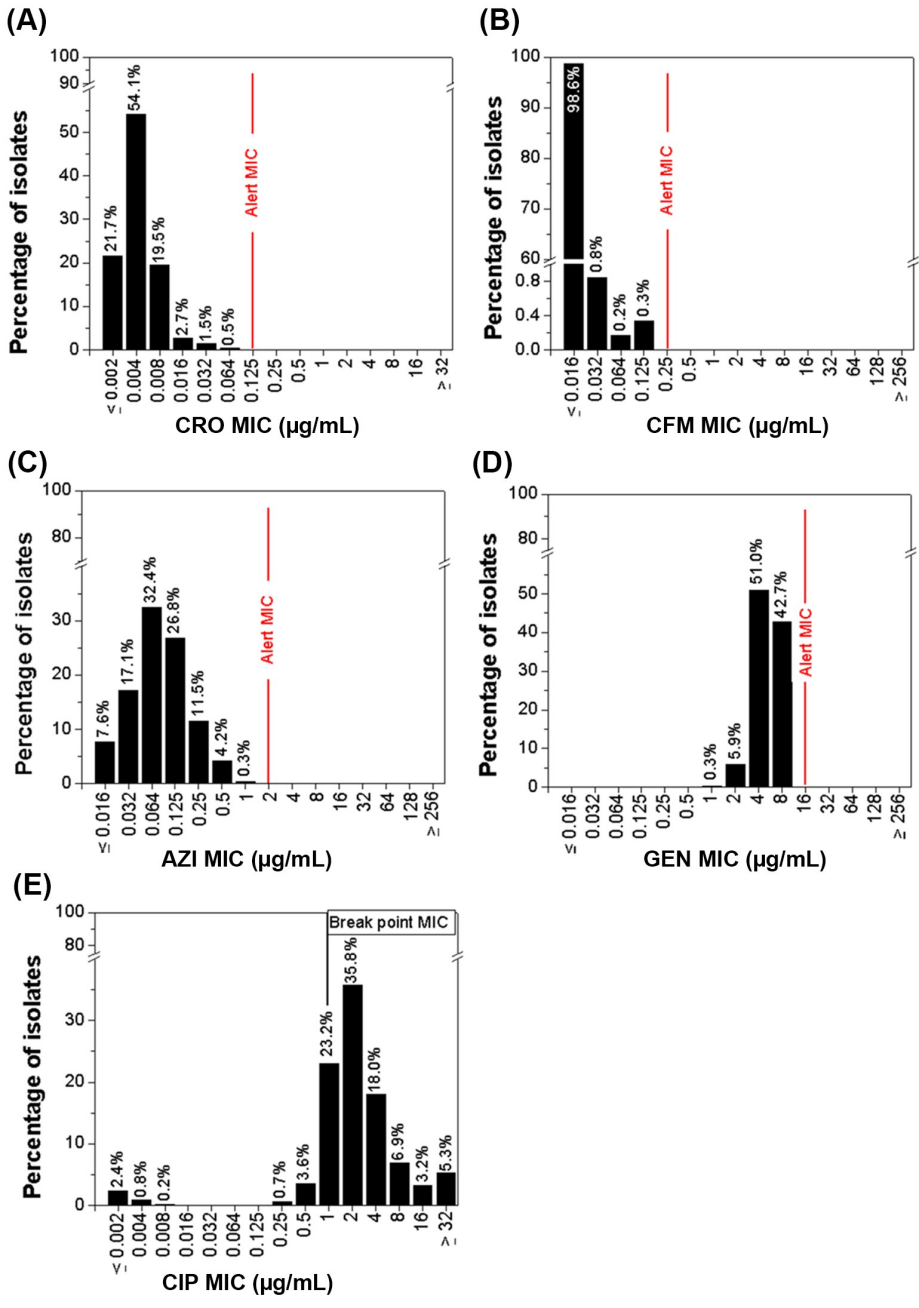
Distribution of MIC values of NG isolates, EGASP Thailand, November 2015 –October 2016 (n = 590). (A), (B), (C), (D), and (E) were Ceftriaxone (CRO), Cefixime (CFM), Azithromycin (AZI), Gentamycin (GEN), and Ciprofloxacin (CIP) Minimum Inhibitory Concentration (MIC) antimicrobial susceptibility test distribution results, respectively.

The distribution of MIC values for the 590 NG isolates are presented for each antimicrobial in Fig 1. The majority of isolates (54.1%) had an MIC value of 0.004 μg/mL to ceftriaxone, 98.6% of isolates had an MIC value of ≤0.016 μg/mL to cefixime, and 32.4% of isolates had an MIC value of 0.064 μg/mL to azithromycin. Among isolates tested to gentamicin, 51.0% had an MIC value of 4.0 μg/mL and 42.7% had an MIC value of 8.0 μg/mL. No isolates had MICs at or above the final alert values for ceftriaxone, cefixime, azithromycin, or gentamicin. Overall, 545 (92.4%) isolates demonstrated resistance to ciprofloxacin with an MIC value ≥1.0 μg/mL.

More than half (65.1%) of the 1,026 men with urethritis had only female sex partners and 41.9% were 18–29 years (Table 2). Most men (97.6%) who went to the GMC clinic at BH had only female sex partners compared to the men that sought care at MHC at BH and SCC @TropMed who had only male sex partners (83.4% and 73.8%, respectively). The age distribution at each clinic was relatively the same; however, men aged 40 years and older primarily went to GMC at BH for services. Overall, 349 (34.0%) men with urethritis had recent antibiotic use in the previous 2 weeks before visiting a participating clinic; more men attending the GMC clinic at BH (39.2%) had recent antibiotic use than men attending other settings.

Of 1,026 men enrolled into EGASP, 564 (55.0%) unique men had NG infection and there were no identified treatment failures. However, among the 564 unique men with NG infection 23 men (4.1%) were diagnosed with a subsequent/repeat NG infection. Among 23 men with repeat NG infection, 20 (87.0%) had an infection diagnosed twice, 2 (8.7%) were diagnosed three times, and 1 (4.3%) was diagnosed more than four times. Men attending the SCC clinic @TropMed had more episodes with more than 1 NG infection (6.2%) compared to men attending a clinic at BH (3.6%). Among men with repeat NG infection, the median time between first and second NG diagnosis was 119 days (range 40–287 days), and the median between second and third NG diagnosis was 132 days (range 110–185 days).

**Table 2:** Characteristics of men with urethritis enrolled in EGASP Thailand, November 2015 –October 2016 (n = 1,026).

Characteristics	N (%)
**Total**	1,026 (100.0)
**Sex of sex partner(s)**	
Men only	293 (28.6)
Women only	668 (65.1)
Men and Women	61 (5.9)
Unknown	4 (0.4)
**Age (years)**	
<18	25 (2.4)
18–29	430 (41.9)
30–39	293 (28.6)
40–49	154 (15.0)
≥50	124 (12.1)
**Recent antibiotic use in past 2 weeks**	
Yes	349 (34.0)
No	674 (65.7)
Unknown	3 (0.3)
**Men with NG infection**	564 (55.0)
Men with ≥1 NG infection*	23 (4.1)

*Percentage is calculated among men with NG infection that were treated (not total number of men enrolled into EGASP).

## Discussion

These are the first data from EGASP, a surveillance system implemented by the Thailand MoPH, WHO and CDC to collect standardized data to monitor for global antimicrobial resistant NG infection. Similar to previously published estimates in the South-East Asia and Western Pacific Region, the vast majority of isolates (92%) in EGASP Thailand were resistant to ciprofloxacin. However, unlike other surveillance systems, all isolates were susceptible to cephalosporins and below an MIC of 2.0 μg/mL for azithromycin. Repeat NG infections occurred in 23 (4%) men; continued efforts to monitor repeat NG infection could support detection of persistent infections, NG treatment failures, and ultimately identify antimicrobial-resistant gonorrhea. Although no cephalosporin-resistant NG isolates were detected, this activity is timely given the emergence of cephalosporin resistance may occur first in South-East Asia [19, 20]. To monitor for emergent antimicrobial-resistant NG and detect early, continuation of EGASP Thailand, and expansion of EGASP to additional global settings, is critical.

Surveillance for antimicrobial-resistant NG with a focus on men with urethritis is appropriate given the need for an augmented global system that can be sustained through sentinel surveillance. MSM are at risk for STIs and in some settings, emergence of AMR occurred first in this population [38]. There was a high proportion of NG infection among MSM in EGASP Thailand, consistent with other studies in Thailand [39], as well as globally [38, 40–42].

There are a number of surveillance systems monitoring for antimicrobial resistant NG in other global settings, including WHO GASP [22], GISP in the U.S. [29], GRASP in the U.K. [30], and AGSP in Australia [31]; each of these surveillance systems have a number of strengths and limitations. The methods for NG identification and AST vary between the surveillance systems as well as the methods for collection of specimens and for monitoring QA/QC processes. Most systems, except WHO GASP, collect some demographic and behavioral data; however, the variables collected across systems are not the same. One challenge with assessing and tracking global antimicrobial resistant NG is the lack of standardized methods across different settings. While WHO GASP is a well-established global surveillance system and is feasible in many settings, it does not collect information on unique individuals, have standardized laboratory methods (e.g., not all settings provide data on MIC values or detect AMR in the same way), or collect demographic, behavioral or clinical information. Core epidemiologic data could be used to support development of effective interventions to prevent the spread of AMR in global settings. EGASP was established to address the limitations of global systems by including standardized demographic, behavioral, clinical and laboratory data, and augmenting surveillance with quality assurance and control measures.

Despite differences in methods for NG surveillance systems globally, it is important to compare EGASP MIC results to those from other systems. The absence of any isolates with final alerts to ceftriaxone, cefixime and azithromycin is lower than published data from other surveillance systems [29–31], however using an MIC of 0.03 μg/mL as a threshold of reduced susceptibility to ceftriaxone according to previous studies [43–46], 2% of NG isolates met this threshold. Overall, 545 (92.4%) isolates demonstrated resistance to ciprofloxacin with an MIC value ≥1.0 μg/mL. This is similar to reports from other countries in the Southeast Asia and Western Pacific regions but is higher than other parts of the world [22, 29]. This distribution of gentamicin MICs is similar to that of previously published reports from other surveillance systems [47]

There was strict attention to the quality of clinical and laboratory data in EGASP Thailand. Although the BH and SCC @TropMed surveillance sites differed in methods of data collection (i.e., paper versus electronic medical charts), the core variables collected between the two surveillance sites were the same and sites could expand on this collection, if desired. Methods for specimen collection, including Gram’s stain, NG culture, identification, and AST were standardized across both surveillance sites. Quality control steps, such as a training and a biannual external quality assessment, were implemented to ensure laboratory quality. Additionally, EGASP Thailand conducted an internal quality assessment (IQA) evaluation in September 2016 to assess data quality [48]. Data from the original source documents (i.e., the medical records and laboratory record books), were compared to the electronic EGASP database as well as the EGASP abstraction form. Clinic staff from each surveillance site conducted the IQA review of the other setting with 10% of cases randomly selected for review. The IQA allowed surveillance personnel to implement improvement measures, including the use of standardized reporting forms, improved systems to prevent data entry errors, and a refresher training to all staff involved in EGASP. The standardization of methods, laboratory activities and assessments, as well as the internal evaluation, were important to increase the reliability of the surveillance data collected.

One unique attribute of EGASP Thailand was the ability to track and evaluate individual men with NG infection and identify those who had repeat infections. Repeat infections are likely re-infections, but they also could be a sentinel event indicating a persistent infection and possible treatment failure, which could suggest antimicrobial-resistant NG infection. Both tests for cure, and evaluation of repeat infections, might support early detection of treatment failure and support a response plan that includes contact tracing and other public health control steps. Many low- and middle-income settings have limited partner notification systems, but a focused effort on potential treatment failures may be feasible. Because most antimicrobial-resistant NG surveillance is based on specimens alone, the opportunity to find early treatment failure is limited to those that have evidence of *in vitro* resistance. Taking steps to have unique patient identifiers connected to NG isolates in a surveillance system is important to assess attributes of persons with antimicrobial-resistant NG, evaluate for clearance of infection, and implement control steps early in the case of treatment failure.

There are a number of limitations of EGASP. We evaluated only men with urethritis with a urethral specimen, in two clinical settings in Bangkok, Thailand; these data may not be generalizable. However, the collection of data in symptomatic men was purposeful as this population provides a high yield for culture. Depending on the global setting, inclusion of other clinical settings, populations at risk, and anatomic sites, are important to consider. While culture capacity is critical to provide susceptibility data, culture has lower sensitivity than nucleic acid amplification testing; therefore, the number of men with gonorrhea in our surveillance system may have been underestimated. Additionally, the inclusion of specimens from the oropharynx and rectum could be beneficial; especially as some studies have detected that AMR might occur more frequently in the oropharynx [9, 13, 49–53]. Lastly, we collected limited demographic, behavioral, and co-infection information; surveillance sites might be able to consider augmenting the core variables, depending on their setting, to include additional risk information for their populations.

In summary, EGASP was established by WHO and CDC to collect standardized and systematic data to monitor trends of antimicrobial-resistant NG. Recent reports on cases of antimicrobial resistant NG infection highlight the importance of this surveillance globally [19]. Data collected from the first EGASP site in Thailand will be used to inform future treatment guidelines in Thailand and the region. The expansion of EGASP to different global settings would augment the ability to detect antimicrobial resistant NG and initiate timely prevention and control steps globally.
